# Correlation between *Hox* code and vertebral morphology in archosaurs

**DOI:** 10.1098/rspb.2015.0077

**Published:** 2015-07-07

**Authors:** Christine Böhmer, Oliver W. M. Rauhut, Gert Wörheide

**Affiliations:** 1 Department für Geo- und Umweltwissenschaften und GeoBio-Center, Ludwig-Maximilians-Universität München, Richard-Wagner-Strasse 10, München 80333, Deutschland; 2 SNSB - Bayerische Staatssammlung für Paläontologie und Geologie, Richard-Wagner-Strasse 10, München 80333, Deutschland

**Keywords:** axial skeleton, evolution, sauropodomorph dinosaurs, regulatory genes, phenotypic variation

## Abstract

The relationship between developmental genes and phenotypic variation is of central interest in evolutionary biology. An excellent example is the role of *Hox* genes in the anteroposterior regionalization of the vertebral column in vertebrates. Archosaurs (crocodiles, dinosaurs including birds) are highly variable both in vertebral morphology and number. Nevertheless, functionally equivalent *Hox* genes are active in the axial skeleton during embryonic development, indicating that the morphological variation across taxa is likely owing to modifications in the pattern of *Hox* gene expression. By using geometric morphometrics, we demonstrate a correlation between vertebral *Hox* code and quantifiable vertebral morphology in modern archosaurs, in which the boundaries between morphological subgroups of vertebrae can be linked to anterior *Hox* gene expression boundaries. Our findings reveal homologous units of cervical vertebrae in modern archosaurs, each with their specific *Hox* gene pattern, enabling us to trace these homologies in the extinct sauropodomorph dinosaurs, a group with highly variable vertebral counts. Based on the quantifiable vertebral morphology, this allows us to infer the underlying genetic mechanisms in vertebral evolution in fossils, which represents not only an important case study, but will lead to a better understanding of the origin of morphological disparity in recent archosaur vertebral columns.

## 1. Introduction

The regionalization of the axial skeleton into a cervical, dorsal, sacral and caudal compartments is a key attribute of amniotes, reflecting an enhanced specialization of the vertebral column to perform different functions. The vertebral column and its associated structures, such as ribs, play a large variety of roles in animal functional morphology and physiology, including in breathing, in sustaining the body posture, in locomotion and in food acquisition. Vertebral morphology and number have thus far-reaching consequences for organismal function and ecology. The form of the axial column is suited to accommodate a broad range of functional roles, receiving multiple mechanical stimuli simultaneously [1], resulting in a high variability of anatomical structures across species. Whereas mammalian presacral count and axial regionalization are very conservative [2–5], reptiles, including dinosaurs and birds, display a high variability in vertebral count [6,7].

The total number of postembryonic vertebrae is determined by the process of somitogenesis [8–13]. The rhythmic formation of somites continues until the total species-specific number of transient embryonic segments is reached [12,14–16]. Subsequently, the vertebral precursors differentiate through resegmentation into vertebrae, exhibiting distinct morphologies depending on their position along the anteroposterior body axis [12,14,15,17,18].

Since the pioneering discovery of homeotic genes, intensive work spanning three decades has shown that the specific temporal and spatial expression pattern of highly conserved *Hox* genes mediates the anteroposterior organization and segmentation of all metazoans, including chordates [9,10,19–21].

*Hox* genes are key determinants of vertebral identity [8–12,22–26] and it has been proposed that a unique or highly distinctive *Hox* code expressed in each somite specifies different vertebral morphologies [27]. Vertebral *Hox* codes have been established for actinopterygian fish [28], mammals [22,23], squamates [23,29–31] and birds [23], but not yet fully for reptiles. In crocodiles, only a partial *Hox* code (eight out of 39 *Hox* genes) for the American alligator (*Alligator mississippiensis*) has been proposed so far [32]. Previous analyses have shown that the vertebral *Hox* code in amniotes is highly conserved, and several *Hox* gene expression boundaries can be used as markers for different regions of the axial skeleton [23]. For example, the expression of *Hox-5* and *Hox-6* genes governs the cervico-thoracic transition in a variety of vertebrate species that differ in cervical number [23]. Likewise, *Hox-10* and *Hox-11* paralogues regulate the formation of the lumbosacral boundary in amniotes [33]. The variation in relative vertebral count appears to be owing to modifications in the pattern of the *Hox* gene activity [6,34]. Although the interactions between *Hox* genes, their target genes and respective mechanisms of activity for vertebral specification are complex and not yet fully understood, knockdown and misexpression experiments have further elucidated the important role of *Hox* gene expression in determining proper vertebral morphology [25,33,35–38].

Studies of *Hox* gene expression patterns thus have the potential to reveal homology between vertebrate body plans and constitute an additional set of characters to homologize segments between organisms [23]. Because adult morphological similarity within an individual vertebral column seems to be related to early *Hox* gene expression [39], the study of morphological variation of vertebrae as an expression pattern proxy therefore potentially provides an opportunity to re-examine long-problematic aspects of morphology, such as the establishing of the exact homologies of different body sections in related taxa with varying vertebral counts.

Here, we analysed the *Hox* gene expression in the cervical vertebral column of the Nile crocodile (*Crocodylus niloticus*) in order to complement and extend a previous examination in the alligator. The correlation between anterior *Hox* gene expression limits and quantifiable changes in vertebral morphology is tested in the cervical vertebral column of extant archosaurs. As a result, the correlation observed in modern crocodiles and birds may allow a reconstruction of the vertebral *Hox* code in extinct relatives such as the sauropodomorph dinosaur *Plateosaurus*.

## 2. Material and methods

### (a) *Hox* gene expression analysis

In order to establish the extant phylogenetic bracket for extinct archosaurs [40], we analysed *Hox* gene expression patterns in crocodilians and birds. This approach allows inference of the likelihood of unknown traits in fossils based on conditions in the two closest extant relatives (crocodilian and bird) bracketing extinct members of this clade (dinosaur). Besides a literature survey, whole-mount *in situ* hybridizations (WISH) were performed in order to complete the cervical *Hox* code for crocodilians. Nile crocodile eggs were collected at the crocodile farm ‘La Ferme aux Crocodiles' in Pierrelatte (France). Embryos were harvested after 9–15 days of development, dissected in 1× PBS and fixed overnight in 4% paraformaldehyde at 4°C followed by serial dehydration to 100% ethanol. Hybridization was done using digoxygenin (DIG)-labelled riboprobes for the cervical *Hox* genes (*HoxA-4*, *B-4*, *C-4*, *D-4* and *A-5*, *B-5*, *C-5*). The RNA probes were detected with NBT/BCIP. The applied WISH protocol is based on that described by Hargrave *et al.* [41] with some modifications. Further details are described in the electronic supplementary material. All novel sequences generated in this study have been deposited in the European Nucleotide Archive (http://ebi.ac.uk/ena; accession nos LN809999–LN810008). All alignments used in this study are freely available at OpenDataLMU (http://dx.doi.org/10.5282/ubm/data68). After WISH, the embryos were sequentially dehydrated into 100% ethanol and photographs were taken immediately with the M165 FC microscope (Leica).

### (b) Morphological analysis

**Figure 1.**
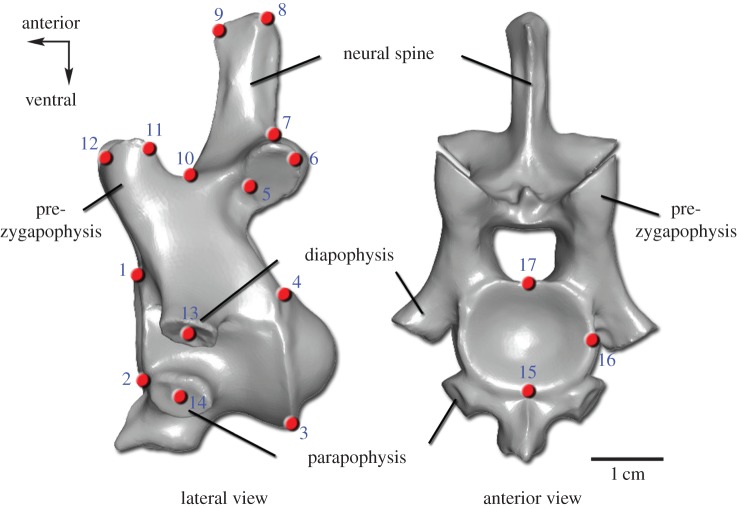
Landmark set used in the geometric morphometric analysis. The numbered three-dimensional landmarks (red points) are shown on the fourth cervical vertebra of *A. mississippiensis* (three-dimensional scan). Detailed definitions of the 17 homologous landmarks are provided in the electronic supplementary material.

Morphological variability within the cervical vertebral column of alligator, crocodile, chicken and the dinosaur *Plateosaurus* was evaluated by a combined morphological analysis. First, qualitative characters were collected and coded as binary or multistate characters. These characters include the presence and absence of osteological features, such as a ventral keel, a bifurcated neural spine and muscle insertion points that vary within each cervical series and could not be captured by homologous landmarks. Second, the morphological differences between the vertebrae within a cervical vertebral column were quantitatively analysed via three-dimensional landmark-based geometric morphometrics. Applying the software Landmark v. 3.0 [42], a series of 17 homologous landmarks were digitized on the three-dimensional scans of the cervical vertebrae (figure 1). The homologous points abstract the vertebral shape and characterize important osteological features, such as the articulation facets of the cervical ribs (diapophysis and parapophysis), which correlate with the corresponding articulating structures of the ribs (tuberculum and capitulum). The first cervical vertebra (atlas) is not included in the geometric morphometric analysis as it is highly modified and lacks specific serial homologies with postatlantal cervicals, and thus, several landmarks cannot be applied to it. The same three-dimensional landmark sets were applied to all analysed taxa in order to provide a comparable basis for the morphological study. Although there are transverse processes connecting the cervical ribs with the vertebral centrum, landmarks (LM) 13 and 14 are not applied in the analysis of chicken because their placement is not exactly repeatable owing to fusion of the ribs to the centra. Geometric morphometric data were processed using the software Morphologika [43] with the following procedures. The coordinates of all landmark sets were superimposed using general procrustes analysis. The Relative Warps (RW) analysis summarized the multi-dimensional information. With the applied settings, this method is equivalent to a principal components analysis. The shape differences were visualized with three-dimensional thin-plate splines. Using the software PAST [44], both datasets were assembled to one data matrix that served as basis for Principal Coordinates (PCO) Analysis. In order to find the similarity relationships among the vertebrae for each taxon, the superimposed three-dimensional landmark coordinates assembled with the qualitative character matrix were analysed with a PCO Analysis applying the Gower index [45,46]. Via the cluster analysis using the single linkage algorithm in combination with the Gower similarity index, the vertebrae were joined based on the smallest distance between them. This resulted in the establishment of morphological subregion patterns of the cervical series for the analysed taxa. Further details are provided in the electronic supplementary material.

### (c) Comparison of genetic and morphological data

To establish phylogenetic homology [47] between *Hox* gene expression in recent archosaurs, we compared *Hox* gene expression patterns in relation to vertebral morphology in crocodiles and birds. Given the sister-taxon relationship of these two groups, the finding that the same *Hox* gene expression boundaries coincide with vertebral subregions is most parsimoniously explained as implying homology between these subregions. These results from recent archosaurs can then be used as a phylogenetic bracket to hypothesize *Hox* gene expression patterns from vertebral morphology in the most recent common ancestor of birds and crocodiles, and in fossil stem-line representatives of these clades [47]. To test the applicability of the results to fossil representatives, we used the basal sauropodomorph dinosaur *Plateosaurus*, as this taxon is represented by numerous, well-preserved skeletons, including complete vertebral columns [48,49].

## 3. Results

### (a) Cervical *Hox* gene expression in the Nile crocodile

**Figure 2.**
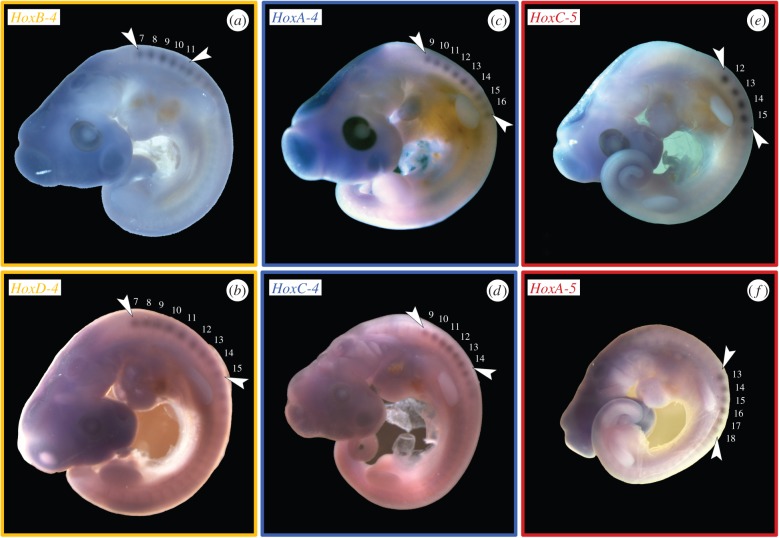
WISH results. *Hox* gene expression in the somites (so) of Nile crocodile embryos (ED 10–14). Arrowheads indicate the anterior and posterior expression boundary. (*a*) *HoxB-4* has an anterior limit at C3 (so 7/8) and extends to C6 (so 10/11). (*b*) *HoxD-4* expression starts at C3 (so 7/8) and fades out posteriorly at D1 (so 14/15). (*c*) *HoxA-4* is expressed from C5 (so 9/10) to D3 (so 16/17). (*d*) *HoxC-4* has an anterior boundary at C5 (so 9/10) and extends to C9 (so 13/14). (*e*) *HoxC-5* expression starts at C8 (so 12/13) and fades out posteriorly at D1 (so 14/15). (*f*) *HoxA-5* is expressed from C9 (so 13/14) to D4 (so 17/18).

The gene expression analysis (figure 2) showed that *C. niloticus* expresses the same *Hox* genes (*HoxA-4*, *B-4*, *C-4*, *D-4* as well as *A-5*, *C-5*) found in the neck of other tetrapods [22,23,29–32,50,51]. In crocodiles, which generally have nine cervicals, the anterior expression limit of *HoxA-4* and *C-4* is at the fifth cervical vertebra (C5), extending to the thoracic region (figure 2*c*,*d*). The expression of *HoxB-4* and *D-4* begins at the third cervical vertebra (figure 2*a*,*b*). *HoxB-4* is only active until C6, whereas *HoxD-4* is expressed to the end of the neck. Whereas the expression of *HoxB-5* already starts at C2 [23], the anterior expression boundary of *HoxA-5* is at the last cervical vertebra (C9) (figure 2*f*). *HoxC-5* is expressed at the last two cervical vertebrae (figure 2*e*).

### (b) Morphological variation within the cervical vertebral column of modern and fossil archosaurs

The analysis of qualitative characters of the vertebrae revealed significant morphological variation within the cervical series in crocodilians and chickens (refer to electronic supplementary material, figure S1). The distribution of osteological features indicates morphological differentiation of the cervical vertebral region in each taxon (refer to electronic supplementary material, tables S1–S3). Although there are minor differences between the Nile crocodile and the American alligator, the variation in qualitative characters indicates four morphological subregions in the crocodilian neck. Five cervical subunits are recognized in the chicken.

**Figure 3.**
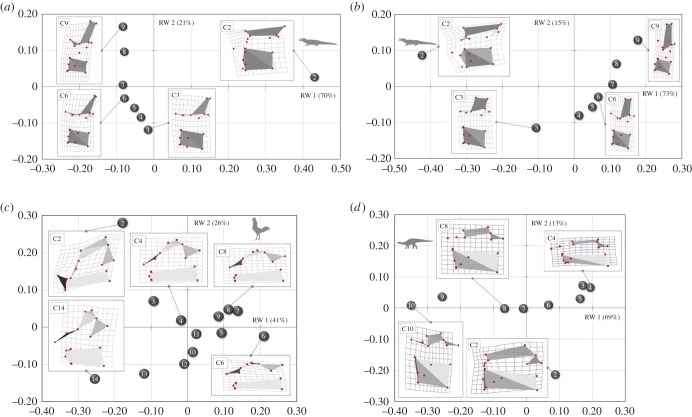
RW analysis results. Each plot shows the shape differences of the cervical vertebrae along RW 1 and RW 2 for (*a*) crocodile, (*b*) alligator, (*c*) chicken and (*d*) *Plateosaurus*. Thin-plate splines (three-dimensional in left lateral view) visualize the variation between landmark configurations of the vertebrae from the respective average shape (zero point).

The landmark-based geometric morphometric study allowed us to quantitatively assess the varying morphology of the cervical vertebrae, to gain additional insights into the regionalization of the neck. The RW analysis summarized the vertebral shape differences and three-dimensional thin-plate splines allowed visualization of the morphological changes from the average (figure 3). The first two RWs explain about 70–90% of the variation in the sample for each examined taxon. In all examined archosaurs, the morphological groups separate along the axes. The morphologically clearly distinct second cervical vertebra always occupies a unique region of the morphospace. A group of following anterior cervicals clusters separately from posterior vertebrae. In between is a cluster of middle cervical vertebrae. In general, the morphological differences within each cervical region involve variation in the shape of the vertebral centrum, the pre- and postzygapophysis and the neural spine. Differences in the relative position of the diapophysis and the parapophysis are only detected in crocodilians, because the vertebrae of the chicken lack unambiguous diapophyseal and parapophyseal landmarks. The morphological differences of the cervical vertebrae, observed along the RW axes, are not a function of size. The size regression analysis (log centroid size versus RWs) revealed no significant correlation between shape variation and size in all analysed taxa.

**Figure 4.**
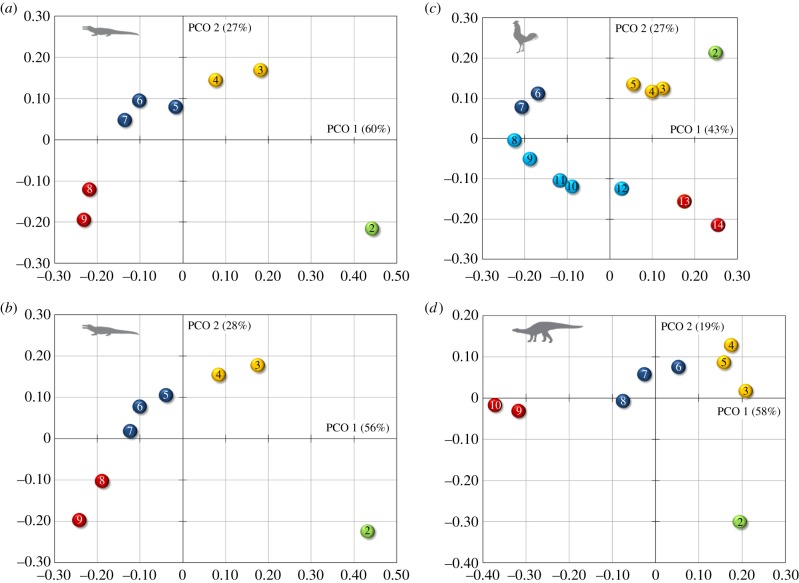
PCO analysis results. The plots for (*a*) crocodile, (*b*) alligator, (*c*) chicken and (*d*) *Plateosaurus* show the discrimination of the cervical vertebrae along PCO 1 and PCO 2. Colours indicate morphological clusters of cervical vertebrae.

Our combined quantitative and qualitative morphological analysis allowed discrimination of vertebrae in different cervical regions (figure 4). The morphospace occupation along the first two PCOs (which account for about 70–90% of the explained variation) for each examined archosaur shows substantial differences among cervical vertebrae. The archosaur neck (excluding the atlas) can be subdivided into 3, 4 or 5 morphological subregions. The general units are the axis complex, an anterior section and a posterior group. The main difference between crocodilians and chicken is an additional subregion that can be recognized in the mid-cervical series in the latter. The analysis recovered four subregions for the crocodilian neck (nine cervical vertebrae), corresponding to the axis, two anterior, three middle and two posterior cervical vertebrae as morphological subregions (figure 4*a*,*b*). The relatively long neck of chicken (14 cervical vertebrae) is subdivided into five morphological subregions (figure 4*c*). Additional to the axis, three anterior, two middle and two posterior cervical vertebrae, there is also a midposterior cervical compartment, comprising C8 to C12. Another difference between the morphological pattern of crocodiles and chickens is that the number of cervical vertebrae that form the anterior subregion is higher and that of the middle subregion is lower in birds. The geometric distance between C2 and C3 is high in the crocodilians and smallest in the chicken (figure 4).

Examination of qualitative characteristics of the vertebrae in the extinct dinosaur *Plateosaurus* (10 cervical vertebrae) revealed distinct morphological differences within the cervical region (see electronic supplementary material, figure S2 and table S4). The distribution of osteological features indicates morphological differentiation of the cervical vertebral region. The regionalization of the dinosaur cervical vertebrae is also indicated by quantitative shape differences, as revealed by landmark analysis (figure 3*d*). The first two RWs explain about 80% of the variation in the vertebrae of *Plateosaurus*. As previously observed for the modern archosaurs, the morphological groups separate along the axes. The morphologically unique second cervical vertebra is distant from the other vertebrae in the morphospace. There is a cluster of anterior cervicals that is separate from the posterior vertebrae. In between, there is a group of middle cervical vertebrae. The morphological differences of the cervical vertebrae in *Plateosaurus*, observed along the RW axes, are not a function of size. The size regression analysis (log centroid size versus RWs) revealed no significant correlation between shape variation and size.

Combining the qualitative and quantitative morphological data via PCA showed substantial differences between the cervical vertebrae of *Plateosaurus*. The morphospace occupation along the first two PCOs (accounting for almost 80% of the explained variation) indicates four morphological subregions in the neck: the axis, three anterior, three middle and two posterior cervical vertebrae (figure 4*d*).

## 4. Discussion

### (a) Cervical *Hox* gene expression in modern archosaurs

**Figure 5.**
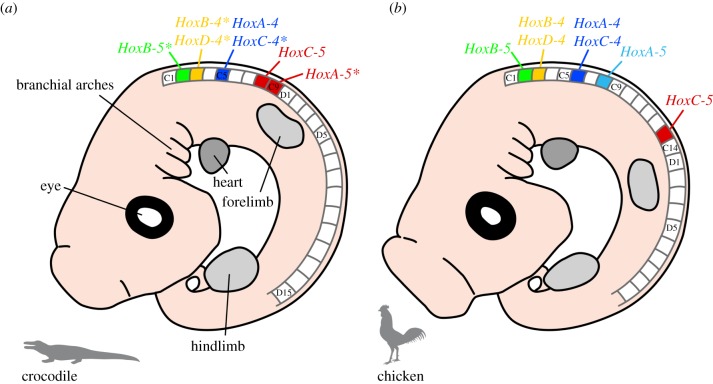
Schematic of the anterior *Hox* expression limits in modern archosaurs. The same *Hox-4* and *-5* paralogues are active in the cervical vertebrae of (*a*) crocodile and (*b*) chicken. In relation to the number of vertebrae, there are differences in the position of the anterior *Hox* expression limits (indicated by colour). Except for *HoxB-5* [32], the *Hox* gene expression analysis in crocodiles was part of this study (figure 2). *Hox* gene data available for alligator [32] are marked with an asterisk. Crocodile and alligator have identical anterior expression limits of *HoxB-4*, *C-4*, *D-4* and *HoxA-5*. It is currently not possible to determine if this correspondence also exists for *HoxB-5* and *C-5* as the exact expression of *HoxB-5* is only known for the alligator and *HoxC-5* has only been investigated in the crocodile. The *Hox* code in chicken is based on references [23,32]. C, cervical; D, dorsal vertebra.

*In situ* hybridization results revealed that the gene expression pattern of *HoxB-4*, *C-4*, *D-4* and *HoxA-5* in the Nile crocodile is identical to that found in the American alligator [32]. The exact expression of *HoxB-5* is only known for the alligator and *HoxC-5* was only investigated in the crocodile. In comparison, the expression of *HoxA-4* and *C-4* in chicken [23], which possess 14 cervical vertebrae, is posteriorly shifted by one vertebra and, thus, begins at the sixth cervical (figure 5). The anterior expression limit of chicken *HoxB-4* and *D-4* is at the second cervical vertebra (axis) and both end at C10 [23]. *HoxA-5* is expressed at the eighth cervical vertebra [32]. It is shifted anteriorly by one vertebra in comparison to the crocodilian pattern. The anterior expression limit of *Hox B-5* is at the axis (C2), as previously observed in crocodilians [32]. The *HoxC-5* expression pattern [23] is also similar to that of crocodiles.

The pattern of cervical expression differs between model species of crocodilians and birds (figure 5). Although the general expression pattern of the *Hox 4* paralogues is relatively similar in all taxa, there is some variation in their anterior expression limits, in relation to the number of cervical vertebrae. The same is seen in the *Hox 5* paralogues, with *HoxA-5* showing the highest variability.

### (b) *Hox* gene expression correlates with morphological subregion pattern in the cervical vertebral column of modern archosaurs

**Figure 6.**
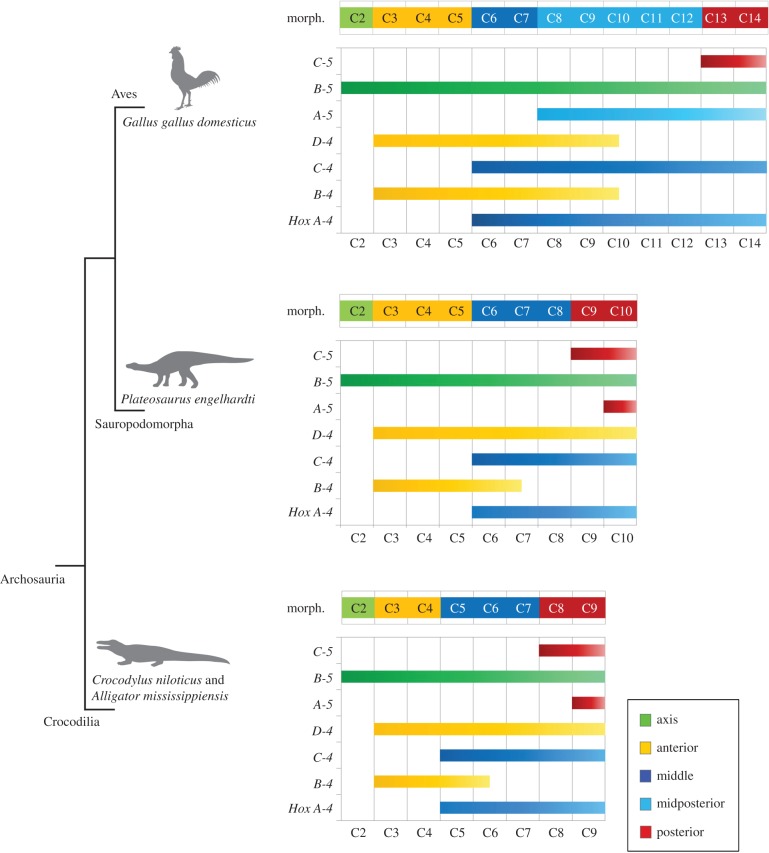
Phylogenetic distribution of morphological subregions and *Hox* gene expression pattern in the neck among extant and extinct archosaurs. The correlation between *Hox* code and vertebral morphology in modern crocodiles and birds allows a reconstruction of the *Hox* code in the extinct archosaur *Plateosaurus* on the basis of the morphological subregion pattern.

The individual vertebral morphology clearly differs between crocodilians and chicken (see electronic supplementary material, figure S1). The combined morphological analysis detected a corresponding pattern of units of vertebrae based on morphological similarity within a cervical series. There is a striking correspondence between anterior expression boundaries of *Hox* genes and distinct vertebral regions along the body axis of modern archosaurs (figure 6). Comparing the *Hox* gene expression pattern with the morphological subregions of the archosaur neck reveals that vertebrae that form clusters in the morphological analysis have identical patterns of *Hox* gene expression. Thus, distinct shape changes in cervical vertebrae (that is between the last vertebra of one subregion and the first vertebra of the following subregion) coincide with differences in the activity of cervical *Hox* genes.

In all analysed archosaurs, the combined morphological analysis revealed that the second cervical vertebra is separate from a group of following anterior cervicals (C3 and C4 in crocodilians; C3, C4 and C5 in chicken) (figure 4). The morphological pattern is reflected in the *Hox* gene expression pattern as the anterior expression boundaries of *HoxB-4* and *D-4* are at the third cervical vertebra in crocodile (this study), alligator [32] and chicken [23,32] (figure 6). Even though it is distinct, the measured shape difference between C2 and C3 in the chicken is not as high as in the crocodilians (figure 4). In contrast to crocodilians, in which the anterior expression boundary of *HoxD-4* is clear (this study, [32]), the published expression of *HoxD-4* in chicken shows a slightly weaker signal and appears to have a relatively unclear anterior boundary [23]. This may be due to inefficient probes. Alternatively, it may indicate a graded anterior expression boundary of *HoxD-4*, which may have an influence on the lower degree of shape change between the second and third cervical vertebra in the chicken.

Although there is one discrepancy in crocodilians regarding the association of *HoxA-5* expression and the posterior cervical vertebrae (figure 6), this correlation strongly indicates that changes in segmental organization are driven by changes in function of *Hox* genes. *HoxA-5* is the most variable in its expression compared with the other *Hox* genes expressed in the cervical vertebral column of the crocodile (this study), alligator and chicken [32]. This may strengthen its previously suggested important role in the patterning of the axial skeleton, which might allow the adaptation to varying functions of the vertebral column in different animals [25,32]. In contrast to both, crocodile and alligator, the expression of *HoxA-5* is anteriorly shifted in the chicken and correlates with the morphological midposterior subregion of the avian neck (figure 6).

In summary, changes in the number of vertebrae are associated with changes in the morphological grouping of the cervical region. The *Hox* gene expression pattern significantly correlates with the pattern of the morphological subregions in the neck of the analysed archosaur taxa. The expression of each *Hox* gene maintains a definite, gene-specific anterior limit that is associated with distinct shape changes within a cervical vertebral column.

### (c) Modification of *Hox* gene expression associated with vertebral evolution

Based on the above, the morphological pattern may serve as a proxy for the underlying *Hox* code in taxa where the genetic information is not available, especially in fossils. Based on these results, and applying the concept of the extant phylogenetic bracket [40], it should be possible to hypothesize changes in the *Hox* code responsible for modifications during vertebral evolution from morphological data, including fossil taxa such as the basal sauropodomorph dinosaur *Plateosaurus*.

In *Plateosaurus*, our analyses showed four morphological subregions in the neck, as seen in crocodilians, but with the difference that the anterior cervical subregion is expanded by one vertebra, as observed in the chicken (figure 4). On the basis of the correlation noted above, a hypothetical *Hox* code for the extinct dinosaur can be established (figure 6). Our results indicate that the posterior shift of the expression boundary of *HoxA-4* and *HoxC-4* seen in modern birds is already present in this basal saurischian dinosaur, whereas the anterior shift of the expression boundary of *HoxA-5* cannot yet be recognized. The morphological results for *Plateosaurus* thus display an intermediate state between the representatives of the extant phylogenetic bracket.

This study showed that the anterior *Hox* gene expression limits shift together with the displacement of cervical subregions. This provides a likely mechanism to explain evolutionary changes along the axial column.

Applying the concept of the extant phylogenetic bracket, and considering that the increase of cervical vertebral number is an evolutionary novelty in birds, the ancestral archosaur *Hox* code was probably similar to that of crocodilians. The general morphological groups of the neck in archosaurs include: the atlas–axis complex, which is specialized for facilitating mobility of the head, the anterior and the middle subregion, and the posterior subgroup, which forms the junction of the highly mobile cervical column to the relatively stiff thoracic column. With the evolutionary elongation of the neck, the *Hox* gene expression patterns were expanded and shifted in relation to each other, respectively. The first step towards the elongation of the cervical vertebral column as seen in chicken in comparison to crocodiles may have been the addition of one vertebra to the anterior section of the neck, as indicated by the presence of this shift, but not other modifications, in the basal dinosaur *Plateosaurus*. The next step may have involved the further addition of vertebrae to the middle region as a result of an expanded *Hox* gene expression domain. Changes in the length of cervical vertebral column associated with changes in the morphological subregions of the neck suggest that important modifications in the expression of *Hox* genes have occurred during archosaur evolution. Among other aspects, this may have facilitated the extraordinary evolution of extremely long necks with up to 19 cervical vertebrae in the sauropodomorph dinosaurs that remain unsurpassed in all other terrestrial animals [52].

## 5. Conclusion

Determination of the number and morphological identity of vertebrae is of major importance in interpreting the evolution of amniotes. The highly conserved *Hox* genes play a fundamental role in the development of the axial column, because they mediate specification of vertebral shape and thus are responsible for the regionalization of the primary body axis.

The WISH experiments revealed that the same *Ho*x-*4* and *Hox-5* paralogue genes are active in the cervical columns of recent archosaurs, exemplified by crocodilians and chicken. By comparing the anterior expression boundaries of the *Hox* genes in modern archosaurs, a correlation between the *Hox* gene expression limits and the boundaries of morphologically distinct subregions within each cervical column is found. Neck elongation is a prominent feature in the evolution of ornithodiran archosaurs, both on the lineage towards modern birds and also in sauropodomorph dinosaurs, in which the extremely elongated neck has been directly linked to their ecological success [52]. On the basis of the results presented here, an evaluation of the importance of modifications in *Hox* gene expression patterns in relation to this neck elongation seems feasible. For the first time, the modifications in *Hox* gene expression in an extinct archosaur, the basal sauropodomorph *Plateosaurus*, were hypothesized with the aim to further our understanding of how evolutionary changes of the axial column might have occurred.

This study shows how the integration of genes, morphology and fossils can improve our understanding of the evolutionary history of modern of modern diversity [53,54]. A more holistic appreciation of vertebral development promises new insights into the evolutionary mechanisms responsible for the great morphological adaptability of the vertebrate axial column. The highly variable cervical region provides an interesting model for the study of the relationship between genomic control and phenotypic changes. The results indicate that the evolution of *Hox* gene expression patterns and associated changes in the axial column is likely to have mediated some of the major transitions in the archosaurian body plan.

## Supplementary Material

Electronic supplementary material

## Data accessibility

All novel sequences generated in this study have been deposited in the European Nucleotide Archive (http://ebi.ac.uk/ena; accession nos LN809999–LN810008). All alignments used in this study are freely available at OpenDataLMU (http://dx.doi.org/10.5282/ubm/data68).

## References

[1] Koob TJ Long JH Jr 2000 The vertebrate body axis: evolution and mechanical function. Am. Zool. 40, 1–18. (10.1668/0003-1569(2000)040[0001:TVBAEA]2.0.CO;2)

[2] Narita Y Kuratani S 2005 Evolution of the vertebral formulae in mammals: a perspective on developmental constraints. J. Exp. Zool. B Mol. Dev. Evol. 304, 91–106. (10.1002/jez.b.21029) 15660398

[3] Galis F 1999 Why do almost all mammals have seven cervical vertebrae? Developmental constraints, *Hox* genes, and cancer. J. Exp. Zool. B Mol. Dev. Evol. 285, 19–26. (10.1002/(SICI)1097-010X(19990415)285:1<19::AID-JEZ3>3.0.CO;2-Z) 10327647

[4] Sánchez-Villagra MR Narita Y Kuratani S 2007 Thoracolumbar vertebral number: the first skeletal synapomorphy for afrotherian mammals. Syst. Biodivers. 5, 1–7. (10.1017/S1477200006002258)

[5] Buchholtz EA Bailin HG Laves SA Yang JT Chan MY Drozd LE 2012 Fixed cervical count and the origin of the mammalian diaphragm. Evol. Dev. 14, 399–411. (10.1111/j.1525-142X.2012.00560.x) 22947313

[6] Müller J Scheyer TM Head JJ Barrett PM Werneburg I Ericson PGP Pol D Sánchez-Villagra MR 2010 Homeotic effects, somitogenesis and the evolution of vertebral numbers in recent and fossil amniotes. Proc. Natl Acad. Sci. USA 107, 2118–2123. (10.1073/pnas.0912622107) 20080660 PMC2836685

[7] Richardson MK Allen SP Wright GM Raynaud A Hanken J 1998 Somite number and vertebrate evolution. Development 125, 151–160. 9486789 10.1242/dev.125.2.151

[8] Wellik DM 2009 *Hox* genes and vertebrate axial pattern. In Hox genes (ed. Pourquie O), pp. 257–278. New York, NY: Academic Press. 10.1016/S0070-2153(09)88009-5 19651308

[9] Carroll SB 1995 Homeotic genes and the evolution of arthropods and chordates. Nature 376, 479–485. (10.1038/376479a0) 7637779

[10] Krumlauf R 1994 *Hox* genes in vertebrate development. Cell 78, 191–201. (10.1016/0092-8674(94)90290-9) 7913880

[11] Kmita M Duboule D 2003 Organizing axes in time and space; 25 years of colinear tinkering. Science 301, 331–333. (10.1126/science.1085753) 12869751

[12] Pourquie O 2003 The segmentation clock: converting embryonic time into spatial pattern. Science 301, 328–330. (10.1126/science.1085887) 12869750

[13] Saga Y Takeda H 2001 The making of the somite: molecular events in vertebrate segmentation. Nat. Rev. Genet. 2, 835–845. (10.1038/35098552) 11715039

[14] Gomez C Pourquie O 2009 Developmental control of segment numbers in vertebrates. J. Exp. Zool. B Mol. Dev. Evol. 312, 533–544. (10.1002/jez.b.21305) 19621429 PMC3094763

[15] Iimura T Pourquie O 2007 *Hox* genes in time and space during vertebrate body formation. Dev. Growth Differ. 49, 265–275. (10.1111/j.1440-169X.2007.00928.x) 17501904

[16] Gomez C Ösbudak EM Wunderlich J Baumann D Lewis J Pourquié O 2008 Control of segment number in vertebrate embryos. Nature 454, 335–339. (10.1038/nature07020) 18563087

[17] Aoyama H Asamoto K 2000 The developmental fate of the rostral/caudal half of a somite for vertebra and rib formation: experimental confirmation of the resegmentation theory using chick–quail chimeras. Mech. Dev. 99, 71–82. (10.1016/S0925-4773(00)00481-0) 11091075

[18] Huang R Zhi Q Brand-Saberi B Christ B 2000 New experimental evidence for somite resegmentation. Anat. Embryol. 202, 195–200. (10.1007/s004290000110) 10994992

[19] McGinnis W Krumlauf R 1992 Homeobox genes and axial patterning. Cell 68, 283–302. (10.1016/0092-8674(92)90471-N) 1346368

[20] Pearson JC Lemons D McGinnis W 2005 Modulating Hox gene functions during animal body patterning. Nat. Rev. Genet. 6, 893–904. (10.1038/nrg1726) 16341070

[21] Wellik DM 2007 *Hox* patterning of the vertebrate axial skeleton. Dev. Dyn. 236, 2454–2463. (10.1002/dvdy.21286) 17685480

[22] Kessel M Gruss P 1990 Murine developmental control genes. Science 249, 374–379. (10.1126/science.1974085) 1974085

[23] Burke AC Nelson CE Morgan BA Tabin C 1995 *Hox* genes and the evolution of vertebrate axial morphology. Development 121, 333–346. 7768176 10.1242/dev.121.2.333

[24] Mallo M Wellik DM Deschamps J 2010 *Hox* genes and regional patterning of vertebrate body plan. Dev. Biol. 344, 7–15. (10.1016/j.ydbio.2010.04.024) 20435029 PMC2909379

[25] Chen JW 2013 Hoxa-5 acts in segmented somites to regulate cervical vertebral morphology. Mech. Dev. 130, 226–240. (10.1016/j.mod.2013.02.002) 23462683

[26] Casaca A Santos AC Mallo M 2014 Controlling Hox gene expression and activity to build the vertebrate axial skeleton. Dev. Dyn. 243, 24–36. (10.1002/dvdy.24007). 23813547

[27] Gaunt SJ 1994 Conservation in the *Hox* code during morphological evolution. Int. J. Dev. Biol. 38, 549–552. 7848839

[28] Morin-Kensicki EM Melancon E Eisen JS 2002 Segmental relationship between somites and vertebral column in zebrafish. Development 129, 3851–3860. 12135923 10.1242/dev.129.16.3851

[29] Cohn MJ Tickle C 1999 Developmental basis of limblessness and axial patterning in snakes. Nature 399, 474–479. (10.1038/20944) 10365960

[30] Woltering JM 2009 Axial patterning in snakes and caecilians: evidence for an alternative interpretation of the *Hox* code. Dev. Biol. 332, 82–89. (10.1016/j.ydbio.2009.04.031) 19409887

[31] Ohya YK Kuraku S Kuratani S 2005 *Hox* code in embryos of Chinese soft-shelled turtle *Pelodiscus sinensis* correlates with the evolutionary innovation in the turtle. J. Exp. Zool. B Mol. Dev. Evol. 304, 107–118. (10.1002/jez.b.21027) 15643629

[32] Mansfield JH Abzhanov A 2010 *Hox* expression in the American alligator and evolution of archosaurian axial patterning. J. Exp. Zool. B Mol. Dev. Evol. 314, 1–16. 20623505 10.1002/jez.b.21364

[33] Wellik DM Capecchi MR 2003 *Hox10* and *Hox11* genes are required to globally pattern the mammalian skeleton. Science 30, 363–367. (10.1126/science.1085672) 12869760

[34] Head JJ Polly PD 2015 Evolution of the snake body form reveals homoplasy in amniote *Hox* gene function. Nature 520, 86–89. (10.1038/nature14042) 25539083

[35] Carapuço M Nóvoa A Bobola N Mallo M 2005 *Hox* genes specify vertebral types in the presomitic mesoderm. Genes Dev. 19, 2116–2121. (10.1101/gad.338705) 16166377 PMC1221883

[36] Horan GS Wu K Wolgemuth DJ Behringer RR 1994 Homeotic transformation of cervical vertebrae in *Hoxa-4* mutant mice. Proc. Natl Acad. Sci. USA 91, 12 644–12 648. (10.1073/pnas.91.26.12644) PMC45495 7809093

[37] Soshnikova N 2014 Hox genes regulation in vertebrates. Dev. Dyn. 243, 49–58. (10.1002/dvdy.24014) 23832853

[38] McIntyre DC Rakshit S Yallowitz AR Loken L Jeannotte L Capecchi MR Wellik DM 2007 Hox patterning of the vertebrate rib cage. Development 134, 2981–2989. (10.1242/Dev.007567) 17626057

[39] Johnson DR O'Higgins P 1996 Is there a link between changes in the vertebral ‘*hox* code’ and the shape of vertebrae? A quantitative study of shape change in the cervical vertebral column of mice. J. Theor. Biol. 183, 89–93. (10.1006/jtbi.1996.0204) 8959111

[40] Witmer LM 1995 The extant phylogenetic bracket and the importance of reconstructing soft tissues in fossils. In Functional morphology in vertebrate paleontology (ed. Thomason J), pp. 19–33. Cambridge, UK: Cambridge University Press.

[41] Hargrave M Bowles J Koopman P 2006 In situ hybridization of whole-mount embryos. In In situ hybridization protocols (eds Darby IA Hewitson TD), pp. 103–113. Totowa, NJ: Humana Press Inc.

[42] Wiley DF 2005 Landmark. 3.0 ed. (http://graphics.idav.ucdavis.edu/research/EvoMorph) University of California, Davis, Institute for Data Analysis and Visualization (IDAV).

[43] O'Higgins P Jones N 2006 Morphologika2. 2.5 ed. (http://hyms.fme.googlepages.com/downloadmorphologica) Hull York Medical School.

[44] Hammer Ø Harper DAT Ryan PD 2001 PAST: palaeontological statistics software package for education and data analysis. Palaeontol. Electron. 4, 1–9.

[45] Gower JC 1966 Some distance properties of latent root and vector methods used in multivariate analysis. Biometrika 53, 325–338. (10.2307/2333639)

[46] Gower JC 1971 A general coefficient of similarity and some of its properties. Biometrics 27, 857–871. (10.2307/2528823)

[47] Nixon KC Carpenter JM 2012 On homology. Cladistics 28, 160–169. (10.1111/j.1096-0031.2011.00371.x) 34861754

[48] Huene FV 1926 Vollständige Osteologie eines Plateosauriden aus dem schwäbischen Keuper. Geol. Paläontol. Abhandlungen 15, 1–43.

[49] Galton PM Upchurch P 2004 Prosauropoda. In The dinosauria (eds Weishampel DB Dodson P Osmólska H), pp. 232–258. Berkeley, CA: University of California Press.

[50] Gaunt SJ Krumlauf R Duboule D 1989 Mouse homeo-genes within a subfamily, Hox-1.4, -2.6 and -5.1, display similar anteroposterior domains of expression in the embryo, but show stage- and tissue-dependent differences in their regulation. Development 107, 131–141. 2576400 10.1242/dev.107.1.131

[51] Rancourt DE Tsuzuki T Capecchi MR 1995 Genetic interaction between hoxb-5 and hoxb-6 is revealed by nonallelic noncomplementation. Genes Dev. 9, 108–122. (10.1101/gad.9.1.108) 7828847

[52] Sander PM 2011 Biology of the sauropod dinosaurs: the evolution of gigantism. Biol. Rev. 86, 117–155. (10.1111/j.1469-185X.2010.00137.x) 21251189 PMC3045712

[53] Slater GJ Harmon LJ Alfaro ME 2012 Integrating fossils with molecular phylogenies improves inference of trait evolution. Evolution 66, 3931–3944. (10.1111/j.1558-5646.2012.01723.x) 23206147

[54] Thewissen JGM Cooper LN Behringer RR 2012 Developmental biology enriches paleontology. J. Verteb. Paleontol. 32, 1223–1234. (10.1080/02724634.2012.707717)

