# Supplementary material for: Correlation between Hox code and vertebral morphology in archosaurs
Source: Proc Biol Sci. 2015 Jul 7;282(1810):20150077. doi: 10.1098/rspb.2015.0077 (PMC4590465; doi:10.1098/rspb.2015.0077)
Supplement: Electronic supplementary material [file rspb20150077supp1.pdf]

ELECTRONIC SUPPLEMENTARY MATERIAL

Correlation between *Hox* code and vertebral morphology in archosaurs: implications for vertebral evolution.

Christine Böhmer, Oliver W. M. Rauhut, Gert Wörheide

1. **Supplementary methodological details:** Whole-mount *in situ* hybridization (WISH).
2. **Supplementary methodological details:** Morphological data.
3. **Supplementary figures:**
   Figure S1. Qualitative morphology of representative cervical vertebrae in extant archosaurs.
   Figure S2. Qualitative morphology of representative cervical vertebrae in the extinct archosaur.
4. **Supplementary tables:**
   Table S1. Qualitative characteristics of the cervical vertebrae of *Crocodylus*.
   Table S2. Qualitative characteristics of the cervical vertebrae of *Alligator.*
   Table S3. Qualitative characteristics of the cervical vertebrae of *Gallus.*
   Table S4. Qualitative characteristics of the cervical vertebrae of *Plateosaurus.*
5. **Supplementary references**

1. Supplementary methodological details: Whole-mount *in situ* hybridization (WISH)

For cDNA synthesis, some embryos were transferred into RNA*later* RNA stabilization reagent after harvesting. Total RNA was extracted using the RNeasy kit from Qiagen according to the manufacturer's instructions.

For the *Hox* genes analyzed in the present study, degenerate primers were designed targeting the conserved 5' region of exon one and the homeobox. The forward (fwd) and reverse (rev) primers were used for amplification of the studied *Hox* genes in the Nile crocodile:

Hox gene	fwd sequence (5'-3')	rev sequence (5'-3')	reference
HoxA-4	GYTCGTTTTTGATAAACTCC	YTTRTGRTCYTTYTTCCAYTTCAT	this study
HoxB-5	TTTTTGATCAACTCCAACTATGT	ATCCTCCTGTTCTGGAACC	[1]
HoxC-4	ATGATCATGAGCTCGTATTTG	ACGGTTTTGGAACCAGATTTTG	[1]
HoxD-4	ATGGCCATGAGTTCGTATATG	GTTCTGAAACCAGATCTTGATC	[1]
HoxA-5	TTTTGTAAACTCATTTTGCG	ATRCTCATRCTTTTCAGC	this study
HoxC-5	GCAGAGCCCCAATATCCCTGCC	TTCATNCKNCKRTTYTGRAACCA	this study, [2]

The primers were designed based on aligned sequences of the specific *Hox* gene available for chicken and mouse (obtained from GenBank database). The intended PCR product size ranged between 400 and 900 bp.

The specific *Hox* gene fragments were amplified from cDNA by polymerase chain reaction (PCR) using 25 uL reaction volumes of GoTaq (Promega). PCR was performed, applying the following cycling parameters: an initial denaturation step at 95°C for 3 min, 35 cycles of 95°C for 30 s, 40°C for 30 s, 72°C for 1 min and a final extension step at 72°C form 5 min. PCR products were purified by standard ammonium acetate-ethanol precipitation and sequenced by applying the BigDye Terminator v3.1 Cycle Sequencing kit (Applied Biosystems). Sequencing was performed in both directions using the same specific *Hox* primers used for PCR. Sequencing reactions were precipitated with sodium acetate-ethanol. Subsequently, the samples were analyzed on the ABI 3730 Genetic Analyzer (Applied Biosystems) at the Sequencing Service of the Department of Biology at the Ludwig-Maximilians-Universität in Munich (Germany). Trace files were assembled in the bioinformatics software suite Geneious [3]. *Hox* gene identity of all obtained sequences was verified using NCBI BLAST [4]. The new sequences were aligned in Geneious with other amniote sequences obtained from GenBank database. All novel sequences generated in this study have been deposited in the European Nucleotide Archive (http://www.ebi.ac.uk/ena; accession numbers #LN809999-#LN810008). All alignments used in this study are freely available at OpenDataLMU (http://dx.doi.org/10.5282/ubm/data68).

After successful identification of the specific *Hox* genes in the Nile crocodile, further PCR reactions under the same conditions, except for a modified reverse primer, were performed to generate antisense-riboprobes for *in situ* hybridization (PCR-based riboprobe synthesis after [5]). For each *Hox* gene, the T3 RNA polymerase promotor sequence (5'-ATTAACCCTCACTAAAGGGA-3') was added to the 5'-end of each gene-specific reverse primer to enable antisense transcripts. From purified cDNA templates, antisense RNA probes were transcribed *in vitro* using T3 RNA polymerase and digoxygenin (DIG)-labelled UTP (Roche).

Hybridization was done using digoxygenin (DIG) -labelled riboprobes for the cervical *Hox* genes (*HoxA-4*, *B-4*, *C-4*, *D-4* and *A-5*, *B-5*, *C-5*). The RNA probes were detected with NBT/BCIP. Embryos stored in ethanol were rehydrated, washed and prehybridized. After prehybridization, DIG-labelled riboprobes were added. The samples were incubated overnight at 60°C. The embryos were stained using anti-DIG fragments coupled to alkaline phosphatase (AP). After washing the color substrates, 4-nitrobluetetrazolium chloride (NBT) and 5-bromo-4-chloro-3-indoyl-phosphate (BCIP) were used to detect the hybridization patterns.

The applied WISH protocol is based on that described by [6] with some modifications. All riboprobes were tested via blot hybridization (in vial).

Day 1

1. Rehydration:
   - Serially rehydration from 100% EtOH into 1x PBS (5 min. at RT).
   - Three additional washes in 1x PBS (5 min. at RT).
2. Proteinase K treatment:
   - Incubation in Proteinase K (1ug/mL) (20 – 30 min at 37°C)
   - Two washes in 1x PBS (5 min at RT).
3. Refixation:
   - Fixation with 4% PFA in 1x PBS (30 min at RT).
   - Five washes in 1x PBS (2x 15 min at RT, 3x 5 min at RT).
4. Prehybridization:
   - Equilibration in 6x SSC (pH 7) (15 min at RT)
   - Incubate in hybridization buffer without riboprobe (2 – 6 h at 60°C) (optional: gently mixing).
5. Hybridization:
   - Incubate in hybridization buffer with denatured riboprobe (overnight at 60°C).

Day 2

6. Post-hybridization washes:
   - Replacing of hybridization buffer with prehybridization buffer from previous step (10 min at 60°C).
   - Six washes in 2x SSC (pH 7) (3x 20 min at RT, 3x 20 min at 60°C).
   - Two washes in 0.1x SSC (pH 7) (30 min at 60°C).
7. Blocking and antibody (detection):
   - Two washes in MAB (10 min at RT).
   - One wash in MAB 2% blocking reagent (1-2 h at RT).
   - One wash in anti-DIG AP in MAB 2% blocking reagent; 1:5000 (overnight at 4°C, rocking).

Day 3

8. Color reaction:
   - Three washes in MAB (1 h at RT).
   - Incubation in 1x Mg-free AP buffer (2x 5 min at RT).
   - Incubation in AP buffer (2x 5 min at RT).

- Monitor color development in color substrate solution (3.3 uL BCIP, 4.4 uL NBT per 1 mL AP buffer) (at 4°C, light-protected).
- Stopping reaction by two washes in MAB and two washes in 1x PBS (5 min at RT).

9. Post-*in situ*:
   - Refixation with 4% PFA in 1x PBS (1-2 h or overnight at 4°C).
   - Five washes in 1x PBS (5 min at RT).

2. Supplementary methodological details: Morphological data

The morphological variability was evaluated by a combined morphological analysis. The modern and fossil taxa analyzed in the present study are listed below. The amniotes differ in number of cervical vertebrae. Cross (†) denotes extinct taxon. Institutional abbreviations: SAPM = Staatliche Sammlung für Anthropologie und Paläoanatomie München, Germany; GPIT = Geologisches und Paläontologisches Institut der Universität Tübingen, Germany; SMNS = Staatliches Museum für Naturkunde Stuttgart, Germany.

Taxon	cervical vertebrae	specimen	3D model
Alligator mississippiensis	9	SAPM #4	X
Alligator mississippiensis		SAPM #3	-
Crocodylus niloticus	9	SAPM #2	X
Gallus gallus domesticus	14	SAPM #133	X
Gallus gallus domesticus		SAPM #134	-
†Plateosaurus engelhardti	10	GPIT/RE/7288	X
†Plateosaurus engelhardti		SMNS 13200	-

First, a set of qualitative characters that vary within each cervical series were collected and coded as binary or multistate characters in a data matrix (Tables S1-S4)Second, the morphological differences between the vertebrae were quantitatively analyzed via landmark-based geometric morphometrics. Three-dimensional scans of the cervical vertebrae of *Gallus gallus* (#133 from SAPM), *Alligator mississippiensis* (#4 from SAPM), *Crocodylus niloticus* (#2 from SAPM) and *Plateosaurus engelhardti* (GPIT/RE/7288) were generated using the laser scanner ModelMaker Z35 integrated with the FaroArm Platinum. The software packages KUBE and Geomagic Studio 9.0 were used for post-processing of the raw data. Applying the software Landmark Version 3.0 [7] a series of 17 homologous landmarks were digitized on the 3D models:

in lateral view:

1. dorsal-anterior edge of the centrum
2. ventral-anterior edge of the centrum
3. ventral-posterior edge of the centrum
4. dorsal-posterior edge of the centrum
5. anteriormost edge of the articular facet of the postzygapophysis
6. dorsal-posterior edge of the articular facet of the postzygapophysis
7. point of maximum curvature between postzygapophysis and neural spine
8. posterior edge of the neural spine
9. anterior edge of the neural spine
10. point of maximum curvature between neural spine and prezygapophysis
11. posteriormost point of the articular facet of the prezygapophysis
12. dorsal-anterior edge of the articular facet of the prezygapophysis
13. center of the diapophysis
14. center of the parapophysis

in anterior view:

15. ventralmost point of the centrum
16. lateralmost point of the centrum
17. dorsalmost point of the centrum

3. Supplementary figures

Figure S1. Qualitative morphology of representative cervical vertebrae in extant archosaurs

The structural features indicate the morphological differentiation of the neck in (A) alligator (SAPM #3) and (B) chicken (SAPM #133). The vertebrae are represented by photographs in left lateral view (first row) and in anterior view (second row). Blue lines and arrows mark important osteological characters (see Tables S2-S3). Note that the dens of C2 is not shown in the alligator.

Abbreviations: caro = processus caroticus, cent = centrum, cond = condylar fossa, crist = cristae laterales, dia = diapophysis, hyp = hypapophysis, keel = ventral keel, para = parapophysis, pneu = pneumatic foramen, poz = postzygapophysis, prez = prezygapophysis, spin = neural spine, tor = torus dorsalis, trans = foramen transversarium, tub = tubercula ansae.

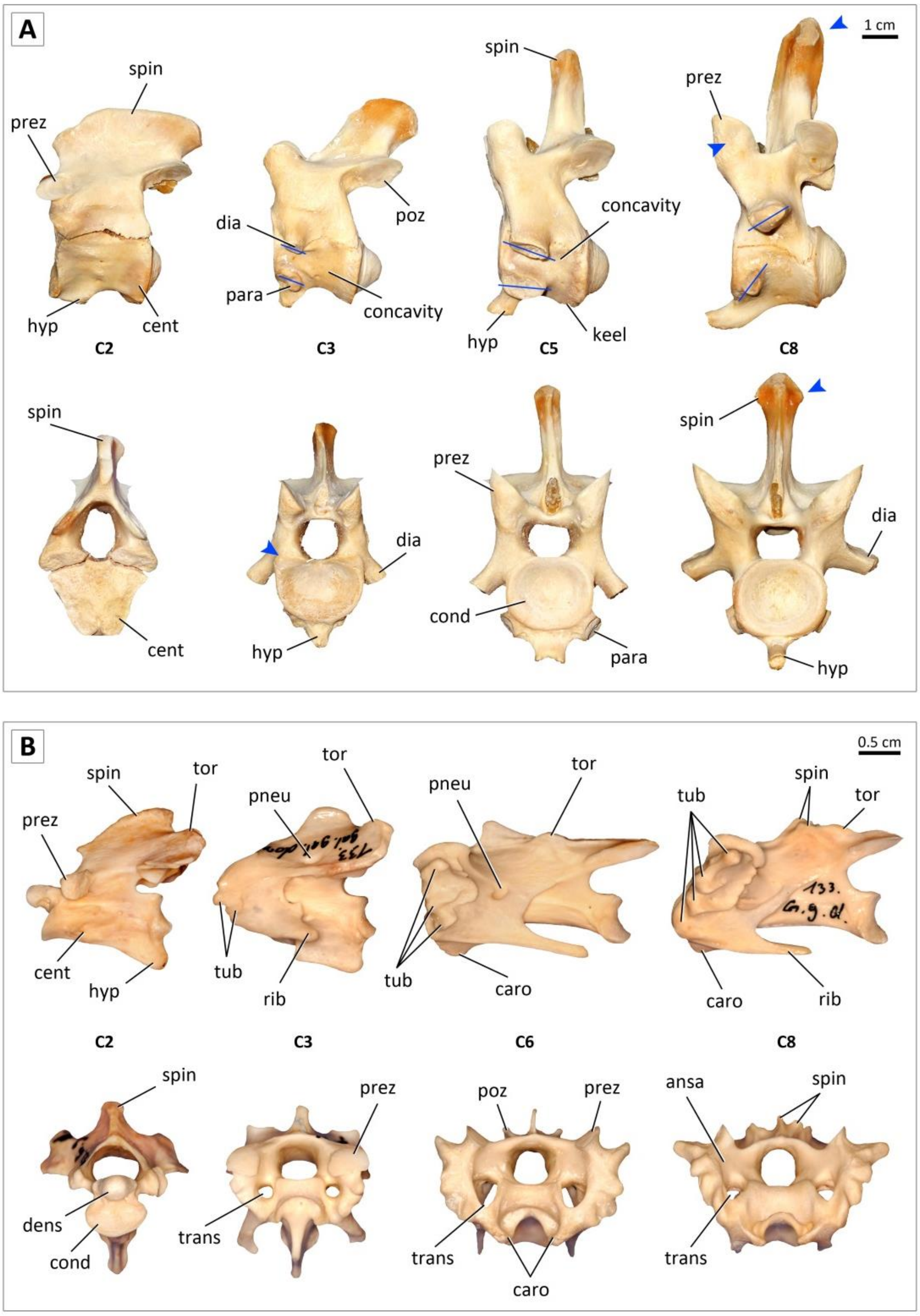

Figure S2. Qualitative morphology of representative cervical vertebrae in the extinct archosaur.

The structural features indicate the morphological differentiation of the neck in Plateosaurus (GPIT/RE/7288). Each vertebra is represented by a photograph in left lateral view and an interpretative line drawing. Details (not to scale) of the 3D model of the respective vertebrae illustrate morphological characters that are not precisely visible in the lateral photographs. Blue lines and arrows mark important osteological characters (see Table S4). Note that the dens of C2 is not shown.

Abbreviations: acdl = anterior centrodiapophyseal lamina, cond = condylar fossa, dia = diapophysis, epi = epipophysis, keel = ventral keel, para = parapophysis, pcdl = posterior centrodiapophyseal lamina, prez = prezygapophysis, spin = neural spine.

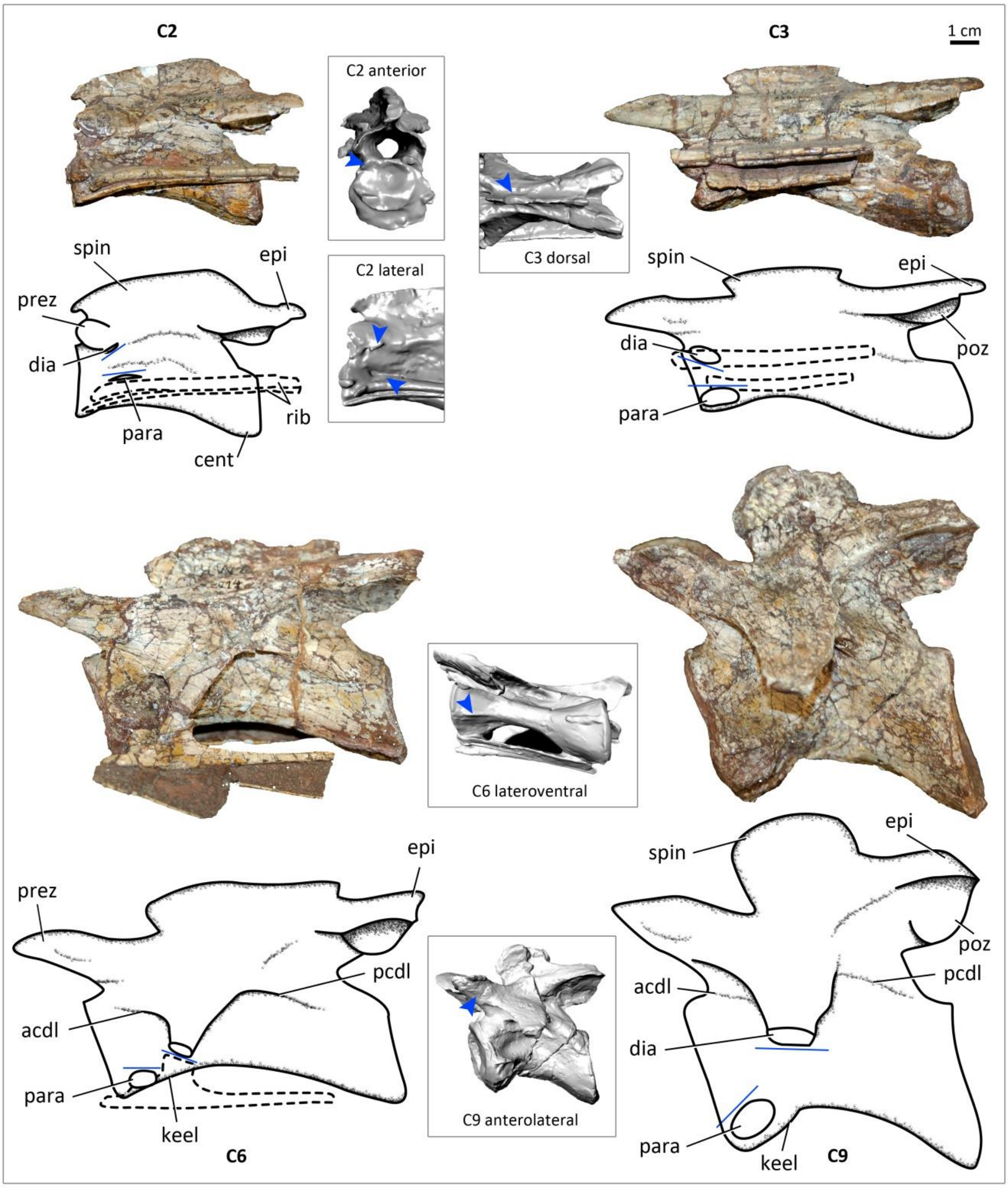

4. Supplementary tables

Table S1. Qualitative characteristics of the cervical vertebrae of *Crocodylus* (specimens: *C. niloticus* SAPM #2, *C. acutus* Field Museum Chicago).

Note that all cervical vertebrae of the Nile crocodile have ribs. It was not possible to identify homologous landmarks on the hypapophysis because its geometry varies considerably from a rounded tubercle (C2) to a truncated process (C3, C4) and an anteriorly pointing, sickle-shaped process (C5 – C9).

character	hypapophysis	neural spine, prominent knob on tip (muscular insertion)	diapophysis, orientation of longitudinal axis	parapophysis; orientation of longitudinal axis	ventral keel extending from hypapophysis, ending in a posterior ventral rugosity on centrum	rugosity on dorsal posterior surface of poz	lateral concavity on centrum	rugosity on latero-dorsal edge of condylar fossa (kidney shape in anterior view)	(rugose) concavity on lateral surface of prez
	1 = rounded<br>2 = truncated<br>3 = pointed, anteriorly	0 = absent<br>1 = present	0 = no axis<br>1 = 45° or less posterior from vertical line<br>2 = 90°/ horizontal	0 = no axis<br>1 = 45° or less posterior from vertical line<br>2 = 90°/ horizontal	0 = absent<br>1 = present	0 = absent<br>1 = present	0 = absent<br>1 = present	0 = absent<br>1 = present	0 = absent<br>1 = present
vertebra									
C2	1	0	0	0	0	1	0	0	0
C3	2	0	0	2	0	1	1	1	0
C4	2	0	2	2	0	1	1	1	0
C5	3	0	2	2	1	1	1	0	1
C6	3	0	2	2	1	1	1	0	1
C7	3	0	2	2	1	1	1	0	1
C8	3	1	1	1	1	0	1	0	1
C9	3	1	1	1	1	0	1	0	1

Table S2. Qualitative characteristics of the cervical vertebrae of *Alligator* (specimens: *A. mississippiensis* SAPM #3, SAPM #4).

Note that all cervical vertebrae of the American alligator have ribs. It was not possible to identify homologous landmarks on the hypapophysis because its geometry varies considerably from a rounded tubercle (C2) to a truncated process (C3, C4) and an anteriorly pointing, sickle-shaped process (C5 – C9).

character	hypapophysis	neural spine, prominent knob on tip (muscular insertion)	diapophysis, orientation of longitudinal axis	parapophysis; orientation of longitudinal axis	ventral keel extending from hypapophysis, ending in a posterior ventral rugosity on centrum	rugosity on dorsal posterior surface of poz	lateral concavity on centrum	rugosity on latero-dorsal edge of condylar fossa (kidney shape in anterior view)	(rugose) concavity on lateral surface of prez
vertebra	1 = rounded<br>2 = truncated<br>3 = pointed, anteriorly	0 = absent<br>1 = present	0 = no axis<br>1 = 45° or less posterior from vertical line<br>2 = 90°/ horizontal<br>3 =315° from vertical line	0 = no axis<br>1 = 45° or less posterior from vertical line<br>2 = 90°/ horizontal<br>3 =315° from vertical line	0 = absent<br>1 = present	0 = absent<br>1 = present	0 = absent<br>1 = present	0 = absent<br>1 = present	0 = absent<br>1 = present
vertebra									
C2	1	0	0	0	0	1	0	0	0
C3	2	0	3	3	0	1	1	1	0
C4	2	0	3	3	0	1	1	1	0
C5	3	0	3	2	1	1	1	0	0
C6	3	0	2	2	1	1	1	0	1
C7	3	0	2	2	1	1	1	0	1
C8	3	1	1	1	1	0	0	0	1
C9	3	1	1	1	1	0	0	0	1

Table S3. Qualitative characteristics of the cervical vertebrae of *Gallus* (specimens: *G. gallus domesticus* SAPM #133, #134, *G. gallus* SAPM # 74, SAPM #103). Note that the cervical vertebrae of chicken have a diapophysis and a parapophysis. However, it was not possible to identify homologous landmarks on these transverse processes because they are completely fused with the cervical ribs. In order to consider the morphology of the Ansa costotransversaria, we analyzed its tubercles and crests.

character	ribs	hypapophysis	bifurcated neural spine	Tuberculae ansa	Cristae laterales	Processus caroticus	Torus dorsalis	Foramen transversarium	pneumatic foramen
vertebra	0 = absent<br>1 = present	0 = absent<br>1 = present	0 = absent<br>1 = present	[0;1;2;3;4] = number	[0;1;2;3;4] = number	0 = absent<br>1 = present	1 = near the tip of poz<br>2 = near the base of neural spine<br>3 = near the base of poz	0 = none<br>1 = round<br>2 = oval	0 = absent<br>1 = present
vertebra									
C2	0	1	0	0	0	0	1	0	0
C3	1	1	0	2	2	0	1	1	1
C4	1	1	0	2	2	0	1	1	1
C5	1	1	0	2	3	0	1	1	0
C6	1	0	0	3	3	1	2	2	1
C7	1	0	0	3	3	1	2	2	1
C8	1	0	1	4	4	1	3	2	1
C9	1	0	1	4	4	1	3	2	1
C10	1	0	1	4	4	1	1	2	1
C11	1	0	1	4	4	1	1	2	1
C12	1	1	1	4	4	0	1	2	1
C13	1	1	1	4	3	0	1	1	0
C14	1	1	1	4	3	0	1	1	0

Table S4. Qualitative characteristics of the cervical vertebrae of *Plateosaurus* (specimens: *P. engelhardti* GPIT/RE/7288, *P. ?engelhardti?* Stuttgart, Berlin)

character	ventral keel on centrum	neural spine, knob on tip (muscular insertion?)	diapophysis, orientation of longitudinal axis	parapophysis; orientation of longitudinal axis	epiphysis	anterior centrodiapophy seal lamina (acdl)	posterior centrodiapophy seal lamina (pcdl)	rugosity on latero-dorsal edge of condylar fossa (kidney shape in anterior view)	(rugose) concavity on lateral surface of prez
vertebra	0 = absent<br>1 = present	0 = absent<br>1 = small<br>2 = prominent	1 = 45° or less posterior from vertical line<br>2 = 90°/ horizontal<br>3 =315° from vertical line	1 = 45° or less posterior from vertical line<br>2 = 90°/ horizontal<br>3 =315° from vertical line	1 = extends over poz<br>2 = does not extend over poz	0 = absent<br>1 = present	0 = absent<br>1 = present	0 = absent<br>1 = present	0 = absent<br>1 = present
vertebra									
C2	0	0	1	2	1	0	0	1	0
C3	0	1	3	2	1	0	0	1	1
C4	0	1	2	2	1	1	1	0	1
C5	0	1	2	2	1	1	1	0	1
C6	1	1	3	2	1	1	1	0	1
C7	1	1	3	2	2	1	1	0	1
C8	1	1	3	2	2	1	1	0	1
C9	1	2	2	1	2	1	1	0	1
C10	1	2	2	1	2	1	1	0	1

5. Supplementary references

1. Mansfield J.H., Abzhanov A. 2010 *Hox* expression in the American alligator and evolution of archosaurian axial patterning. *J. Exp. Zool. B Mol. Dev. Evol.* **314**, 1-16.
2. Liang D., Wu R., Geng J., Wang C., Zhang P. 2011 A general scenario of *Hox* gene inventory variation among major sarcopterygian lineages. *BMC Evol. Biol.* **11**, 25.
3. Drummond A.J., Ashton B., Buxton S., Cheung M., Cooper A., Duran C., Field M., Heled J., Kearse M., Markowitz S., et al. 2011 Geneious R6 (Version 6.0.3) created by Biomatters.
4. Johnson M., Zaretskaya I., Raytselis Y., Merezhuk Y., McGinnis S., Madden T.L. 2008 NCBI BLAST: a better web interface. *Nucleic Acids Res.* **36** (Web Server issue), W5-9. (doi:10.1093/nar/gkn201).
5. David R., Wedlich D. 2001 PCR-based RNA probes: a quick and sensitive method to improve whole mount embryo in situ hybridizations. *BioTechniques* **30**, 769-772, 774.
6. Hargrave M., Bowles J., Koopman P. 2006 In Situ Hybridization of Whole-Mount Embryos. In *In Situ Hybridization Protocols* (eds. Darby I.A., Hewitson T.D.), pp. 103-113. Totowa, Humana Press Inc.
7. Wiley D.F. 2005 Landmark. (3.0 ed. University of California, Davis, Institute for Data Analysis and Visualization (IDAV).
